# Predictors and consequences of long-term pregnancy-related pelvic girdle pain: a longitudinal follow-up study

**DOI:** 10.1186/s12891-016-1154-0

**Published:** 2016-07-12

**Authors:** Helen Elden, Annelie Gutke, Gunilla Kjellby-Wendt, Monika Fagevik-Olsen, Hans-Christian Ostgaard

**Affiliations:** Gothenburg University, Institute of Health and Caring Sciences, Sahlgrenska Academy, S-405 30 Gothenburg, Sweden; Department of Health and Rehabilitation/Physiotherapy, Sahlgrenska Academy, Gothenburg University, Institute of Neuroscience and Physiology, S-405 30 Gothenburg, Sweden; Department of Occupational Therapy and Physical Therapy, Sahlgrenska University Hospital/ Molndal, S-431 80 Molndal Gothenburg, Sweden; Department of Occupational Therapy and Physical Therapy, Sahlgrenska University Hospital, s-413 45 Gothenburg, Sweden; Department of Orthopaedics, Sahlgrenska Academy, Molndal Hospital, S-431 80 Molndal, Sweden

**Keywords:** Anxiety, Depression, Function, Health-related quality of life, Long-term, Pain catastrophizing, Pelvic girdle pain, Predictor, Self-efficacy

## Abstract

**Background:**

Pelvic girdle pain (PGP) is a multifactorial condition, which can be mentally and physically compromising both during and after pregnancy. However, long-term pregnancy-related PGP has been poorly investigated. This longitudinal follow-up study uniquely aimed to describe prevalence and predictors of PGP and its consequences on women’s health and function up to 11 years after pregnancy.

**Methods/Design:**

A postal questionnaire was sent to 530 women who participated in 1 of 3 randomized controlled studies for PGP in pregnancy. Women who reported experiencing lumbopelvic pain were offered a clinical examination. Main outcome measure was the presence of long term PGP as assessed by an independent examiner. Secondary outcomes were: working hours/week, function (the Disability Rating Index, and Oswestry Disability Index), self-efficacy (the General Self-Efficacy Scale), HRQL (Euro-Qol 5D and EQ-Visual scale), anxiety and depression, (Hospital anxiety and depression scale,) and pain-catastrophizing (Pain Catastrophizing Scale), in women with PGP compared to women with no PGP.

**Results:**

A total of 371/530 (70 %) women responded and 37/ 371 (10 %) were classified with long-term PGP. Pregnancy-related predictors for long-term PGP were number of positive pain provocation tests (OR = 1.79), history of low back pain (LBP) (OR = 2.28), positive symphysis pressure test (OR = 2.01), positive Faber (Patrick’s) test (OR = 2.22), and positive modified Trendelenburg test (OR = 2.20). Women with PGP had significantly decreased ability to perform daily activities (*p* < .001), lower self-efficacy (*p* = 0.046), decreased HRQL (*p* < .001), higher levels of anxiety and depression (*p* < .001), were more prone to pain catastrophizing, and worked significantly fewer hours/week (*p* = 0.032) compared to women with no PGP.

**Conclusions:**

This unique long-term follow up of PGP highlights the importance of assessment of pain in the lumbopelvic area early in pregnancy and postpartum in order to identify women with risk of long term pain. One of 10 women with PGP in pregnancy has severe consequences up to 11 years later. They could be identified by number of positive pain provocation tests and experience of previous LBP. Access to evidence based treatments are important for individual and socioeconomic reasons.

## Background

Pelvic girdle pain (PGP) is a multifactorial condition with a partly unknown aetiology [1, 2] PGP can be mentally and physically compromising both during and after pregnancy [2–12]. Previous research reports a prevalence of PGP from the postpartum stage to 3 years after childbirth from 1 to 43 % [13–16], and 7 % at 6 years [17]. Disability and pain intensity in pregnancy are associated with sick leave due to pain in pregnancy and persistent pain [16, 18, 19]. Some studies have focused on prevalence, consequences, risk factors, prognostic factors [2, 9, 13, 16–18, 20–35] and protecting factors while others have evaluated treatment outcomes [36–38]. However, the prevalence predictors and consequences of long-term pregnancy-related PGP have been poorly investigated.

Guidelines for the diagnosis of PGP state that this pain is experienced between the posterior iliac crest and the gluteal fold, particularly in the vicinity of the sacroiliac joints (SIJ), separately or in conjunction with pain in the symphysis, and that pain and functional disturbances in relation to PGP must be reproducible by specific clinical tests [5]. In most studies, however, no examination during pregnancy was performed, and there is no study with more than a 3-year follow-up postpartum that has classified PGP by clinical examination [14]. Women with a pelvic girdle syndrome (PGS), i.e. an anterior and posterior pain location, have been found to have the worst prognosis, and those with isolated anterior, i.e. symphysiolysial pain, the best prognosis [13].

Suggested pregnancy-related predictors for long-term PGP include demographic variables, such as: age [2, 23, 27] and high Body Mass Index (BMI) [16], work-related variables strenuous work [28] and sick leave [29], delivery-related variables e.g. previous caesarean section [21, 26, 30], and higher fetal weight [20], PGP-related variables e.g. previous low back pain (LBP) [2, 31], hypermobility [16], severe pain [2, 16], decreased function [31], ≥8 h sleep or rest/day [20], PGS [13, 32], difficulty in performing the Active Straight Leg Raise test [33], number of positive pain provocation tests [31, 34, 39] and emotional distress [25].

Development from acute to chronic pain is complex, and more research is needed to explore why some women develop long-term PGP after delivery, which women are at greater risk, and whether prevention is possible. To our knowledge, this is the only longitudinal follow-up study describing the prevalence, predictors and consequences of PGP up to 11 years after pregnancy.

## Methods

Data were obtained through one self-administered questionnaire, sent and returned by mail. Two reminders were sent. Presence of lumbopelvic pain (LPP) was assessed by one question, recommended by a modified Delphi study conducted with 28 experts in back pain research from 12 countries [40]. According to the above mentioned recommended question, women were asked whether they had experienced LPP with or without radiation into either or both legs during the past 4 weeks. The pain should have been bad enough to limit usual activities or cause changes in daily routines for more than one day. The questionnaire included information about the follow-up study and contact details for additional information.

### Outcome measures

The primary outcome measure and dependent variable was the presence of long-term PGP.

Secondary outcomes were marital status, educational level, physical activity, employment, working hours/week, ability to take breaks at work, number of pregnancies, parity, caesarean section and birth weight and sex of last born baby, use of hormonal contraceptives, and menopause. Function was measured on the Disability Rating Index (DRI) [41] and the Oswestry Disability Index (ODI) [42]. The DRI [41] measures ability in 12 activities, indicated on a100 mm visual analogue scale (VAS) (where 0 = the ability to perform an activity without difficulty, and 100 = no ability whatsoever to perform the activity) [43]. An index is achieved by measuring the distance in millimeter from 0 to the women’s markings on the VAS. The mean of these measurements provides the DRI expressed in percent of the highest possible rating. Pain when ‘turning in bed’ (where 0 = no pain and 1 = pain) was also queried, since this was performed in the original RCTs [44–46]. Ten different items on perceived disability were rated on the ODI: pain intensity, personal care and lifting, walking, sitting, standing, sleeping, sexual life, social life and travelling. The items were scored from 0 to 5. The scores of all items were added, giving a possible maximum score of 50. The total score was then doubled and expressed as percentages where 0 % represents no disability, 0–20 % no or minimal disability, 20–40 % moderate disability, 40–60 % severe disability, 60–80 % crippled and 80–100 % bed bound. Health-related quality of life (HRQL) was measured with the European quality of life measure (the EuroQol- 5Dimensions (EQ-5D), and the EQ-Visual analogue Scale (EQ-VAS) [47]. The EQ- 5D [47] assesses five dimensions of HRQL: mobility, self-care, activities of daily life, pain. Levels of anxiety and depression were measured. For each dimension, the woman describes three possible levels of problems (none, mild to moderate and severe). This descriptive system contains 243 combinations or index values for state of health. The total score range is from −0.43 to 1.0, in which -0.43 is the lowest health state, and 1, the highest. For a normal population, the average value is 0.8-0.9 [48]. The EQ-5D VAS is a vertical VAS (0–100 in which 0 is the lowest conceivable health state, and 100 the optimal health state) [47]. Levels of anxiety and depression was measured on the Hospital Anxiety and Depression Scale (HADS) [49]. The HADS is a 14-item scale for detection of anxiety and depression in people with physical health problems. Seven items relate to anxiety (HADS-A) and 7 items to depression (HADS-D). Each item on the questionnaire is scored from 0-3, indicating that a person can score between a total of 0 and 21 for either anxiety or depression. A cut-off point of 8/21 for anxiety or depression has been identified [50]. For anxiety this gave a specificity of 0.78, and a sensitivity of 0.9. For depression, this gave a specificity of 0.79, and a sensitivity of 0.83 [50]. Self-efficacy was measured with the General self-efficacy Scale (GES) [51]. The GES [51] is a 9-item scale that measures self-efficacy, and the range is from 10-40 points, a sum score is usually calculated. Pain catastrophizing was measured with the Pain Catastrophizing Scale (PCS). The PCS [52] was developed as a self-report measurement tool that provided a valid index of catastrophizing in clinical and non-clinical populations [52]. The PCS is a 13-item self-report scale to measure thoughts and feelings related to pain (e.g. “when I am in pain, I worry all the time about whether the pain will end”). In the PCS, each item is rated on a 5-point scale: (in which 0 is not at all, and 4, constantly). A total score is calculated (range 0-65 points). The three subscales of magnification, rumination, and helplessness reveal different dimensions of the same underlying content. Women stating LPP were also asked questions about sleep, use of analgesia, sick-leave due to LPP, and severity of LPP in relation to work, and offered an examination.

### Assessment

Women experiencing LPP in the questionnaire were contacted by telephone. During the telephone interview they could confirm the prevalence, location and severity of symptoms, and were asked about exclusion criteria i.e. on-going pregnancy and/or systemic disease. If they fulfilled the criteria and agreed on an examination, they were scheduled to one of two specially trained physiotherapists. A standardised and reliable assessment [53] was performed in order to classify the women’s LPP into the categories PGP, PGP plus LBP, or only LBP. The classification included a history of pain provocation in different positions or activities of daily living, pelvic pain provocations tests, and a standardised mechanical assessment of the lumbar spine according to Mechanical Diagnosis and Therapy [53]. This assessment also included repeated end range flexion and extension movements in standing position and/or in lying position. The pelvic pain provocation tests used were: the posterior pelvic pain provocation test (P4 test) [54], distraction test, compression test, sacral thrust [53] and the MAT-test [55]. The MAT-test: standing with one hip in abduction; perform an adduction simulating the movement to pull a mat. The MAT-test was added as a replacement for the symphysis pubis pressure test [55, 56]. The reason for the decision to use the MAT test instead of the symphysis pubic pressure test was both ethically and scientifically based. The symphysis pubic pressure test provokes severe pain that does not subside directly, and the test has been shown to be false positive in women with no PGP [56]. The MAT test has been shown to have a high percentage of agreement with palpation of the pubic symphysis. [55]. In order to use the same classification used in the original RCTs [44–46]. The Faber (Patrick's) test and modified Trendelenburg test were also performed [57]. A neurological examination was performed if the women had a history of pain radiating into the leg. Load transfer between trunk and pelvis evaluated the active straight leg test (ASLR test) [58]. A classification of PGP was defined according to European guidelines [5]. All of the following criteria had to be fulfilled:

- Pain experienced between the posterior iliac crest and the gluteal fold, particularly in the vicinity of the SIJ in conjunction with/or separately in the symphysis.
- Reports of duration and weight-bearing-related pain in the pelvic girdle.
- Diminished endurance in standing, walking and sitting.
- Positive clinical diagnostic tests, which reproduced pain in the pelvic girdle.
- No nerve root syndrome
- No reproducible pain and/or changed symptoms in the lumbar spine by repeated end range movement.

A classification of LBP was defined as:

- Reported pain in the lumbar spine with or without radiation to the leg.
- Reproducible pain and/or changed symptoms in the lumbar spine by repeated end range motion [53].

A classification of combined PGP and LBP were defined when both of the above criteria were fulfilled. Since the focus of this follow up was to study long-term PGP, women with PGP and women with PGP + LBP are hereafter presented in the same group and named ‘PGP’.

### Severity of PGP

Predictors of PGP were grouped and described in 5 clusters e.g., demographic, work-related, pregnancy-related and PGP-related predictors. The demographic predictor was age at randomisation in the RCT. Work-related predictors included severity of PGP in relation to work at randomisation, ability to take breaks at work, and sick leave due to PGP at randomisation. Pregnancy-related predictors were age at menarche plus total number of pregnancies and parity at the follow-up. PGP-related predictors were: a history of LBP before randomisation, function on the DRI [41], pain when turning in bed, HRQL (EQ-5D and EQ-VAS) [47], pain intensity related to motion in the morning and evening on a 0-100 mm VAS [43], unpleasantness of PGP on a 0-100 mm VAS, the P4 test [54], Faber (Patrick's) test, the symphysis pubis pressure test, the modified Trendelenburg's test [57], and the ASLR test [59] at randomisation. However, EQ-5D, EQ-VAS and the ASLR test were not used in Elden et al. 2005 [44].

The median, CI, quartiles, means and SD were calculated when appropriate. Continuous variables are presented with mean, Standard Deviation (SD) and 95 % Confidence Interval or median, minimum and maximum, and categorical variables are presented with number and percentage. For comparison between groups Fisher´s Exact test was used for dichotomous variables, Mantel-Haenszel’s Chi Square Exact test for ordered categorical variables, Chi Square test for non-ordered categorical variables and the Mann-Whitney *U*-test were used for continuous variables. In order to find independent predictors to occurrence of PGP, all significant variables from the univariable step were entered into a stepwise multivariable forward logistic regression. *P* < 0.10 was the prerequisite entry in the stepwise model. The results of the logistic regression analysis are presented as Odds Ratio (OR) with a 95 % confidence interval for each predictor and as the area under the ROC curve (AUC) for the strongest predictor. All significance tests were two-sided and conducted at the 5 % significance level. All statistical analysis was performed with SAS System version 9, SAS Institute, Cary, NC, USA.

## Results

**Fig. 1:**
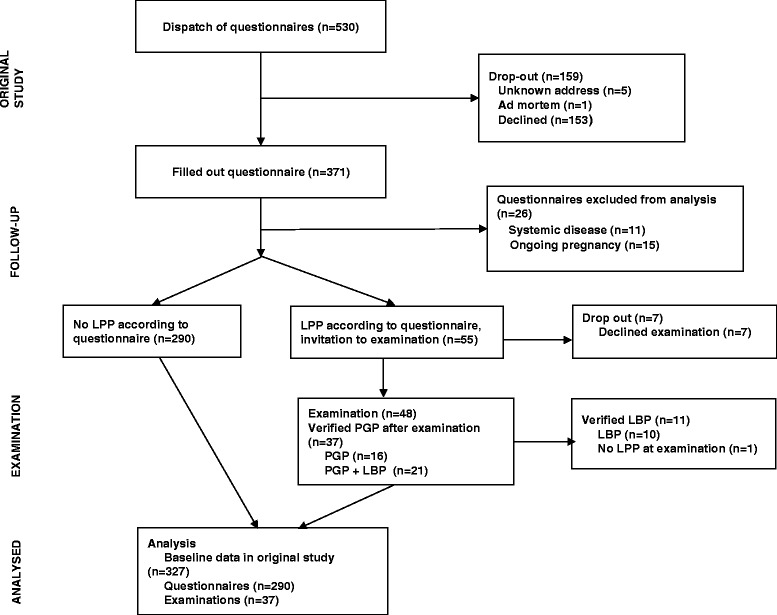
Flow-chart of the study

Of 536 women in one of the three original studies, only 530 questionnaires were sent out, since six women had participated in two of the RCTs. A total of 371 (70 %) responded to the questionnaire. However 11 women were excluded due to systemic disease and 15 due to on-going pregnancy. Thirty-seven/345 (10.7 %) women were classified with PGP. The clinical classification showed that 16 women suffered from PGP, and 21 women from PGP plus LBP. Among the women with PGP; two women had only an anterior pain location i.e. symphysiolysis; nine had a posterior pain location; and five had both anterior and posterior pain compared to women “with” no PGP at follow up. Figure 1 shows the progress of participants throughout the study, and the results of the examination at follow-up.

Table 1 shows the baseline characteristic of the study population by PGP and no PGP. More women with persistent PGP had previous LBP and the women had a higher number of positive pain provocation tests compared to women with no PGP at follow-up.

**Table 1:** Characteristics of women with PGP or PGP plus LBP and women with no PGP before inclusion in RCT in pregnancy

Variable	PGP or PGP + LBP (*n* = 37)	No PGP (*n* = 290)	*p*-value
Treatment in RCT			
Standard treatment	11 (29.7 %)	77 (26.6 %)	
Standard treatment + Acupuncture	14 (37.8 %)	129 (44.5 %)	
Standard treatment + Specific stabilising exercises	4 (10.8 %)	56 (19.3 %)	
Standard treatment + Craniosacral therapy	8 (21.6 %)	28 (9.7 %)	0.11
Age, years	30.0 (23.0; 39.0)	31.0 (20.0; 43.0)	0.40
	*n* = 37	*n* = 289	
BMI before pregnancy	22.6 (19.7; 34.2)	23.2 (18.0; 38.4)	0.99
	*n* = 23	*n* = 115	
Age at menarche, years	13.0 (10.0; 15.0)	13.0 (9.0; 16.0)	0.63
	*n* = 32	*n* = 248	
Previous LBP	24 (64.9 %)	127 (44.7 %)	0.032
Women on sick-leave due to PGP	13 (35.1 %)	143 (50.0 %)	0.13
Severity of PGP			
No complaints, PGP do not affect ability to work	1 (2.7 %)	5 (1.8 %)	
Moderate complaints, PGP only affect ability to work sporadically	5 (13.5 %)	67 (23.8 %)	
Not insignificant, cannot do some parts of my work	15 (40.5 %)	90 (32.0 %)	
Severe, can almost not work	12 (32.4 %)	79 (28.1 %)	
Severe, cannot work at all	4 (10.8 %)	40 (14.2 %)	0.80
Tests for assessment of PGP before inclusion in the RCT			
Pain provocation tests			
P4 test	37 (100.0 %)	283 (97.6 %)	0.86
Symphysis pressure test	22 (59.5 %)	121 (42.2 %)	0.070
Patrick Faber test	27 (73.0 %)	159 (54.8 %)	0.051
Modified Trendelenburg test	22 (59.5 %)	116 (40.0 %)	0.039
Number of bilateral positive pain provocation tests			
0	0 (0.0 %)	3 (1.0 %)	
1	6 (16.2 %)	58 (20.0 %)	
2	6 (16.2 %)	107 (36.9 %)	
3	10 (27.0 %)	81 (27.9 %)	
4	15 (40.5 %)	41 (14.1 %)	0.0013
Functional test			
ASLR test (sum of scores)	3.00 (0.00; 8.00)	3.00 (0.00; 10.00)	0.35
	*n* = 23	*n* = 116	
Subgroups of pelvic girdle pain			
Solely symphysiolysis	0 (0.0 %)	5 (1.7 %)	1.00
One sided sacroiliac pain	3 (8.1 %)	37 (12.8 %)	0.61
One sided sacroiliac pain + symphyseal pain	6 (16.2 %)	39 (13.4 %)	0.80
Double sided sacroiliac pain	12 (32.4 %)	118 (40.7 %)	0.43
Pelvic girdle syndrome	16 (43.2 %)	91 (31.4 %)	0.21
Pain related to motion			
In the morning, VAS	31.0 (8.0; 92.0)	26.5 (0.0; 96.0	0.089
In the evening, VAS	62.0 (5.0; 93.0)	62.8 (6.0; 100.0)	0.30
	*n* = 37	*n* = 288	
Unpleasantness of PGP, VAS	63.0 (20.0; 100.0)	73.0 (0.0; 100.0)	0.068
	*n* = 30	*n* = 200	
DRI	50.0 (23.0; 100.0)	59.0 (11.0; 100.0)	0.11
	*n* = 37	*n* = 279	
EQ-VAS	40.0 (25.0; 100.0)	50.0 (20.0; 99.0)	0.37
	*n* = 23	*n* = 113	
EQ-5D score	0.620 (-0.016; 0.760)	0.620 (-0.074; 0.796)	0.23
	*n* = 23	*n* = 112	
Education level			
Primary school	0 (0.0 %)	5 (2.0 %)	
Secondary school	11 (33.3 %)	64 (25.3 %)	
College	5 (15.2 %)	21 (8.3 %)	
University degree	17 (51.5 %)	163 (64.4 %)	0.37
No or rare ability to take rest breaks at work	4 (13.8 %)	68 (29.8 %)	0.18
Physical activity ≥30 minutes during leisure before pregnancy, days/week			
0	3 (9.1 %)	8 (3.2 %)	
1	1 (3.0 %)	20 (7.9 %)	
2	3 (9.1 %)	43 (17.1 %)	
3	7 (21.2 %)	57 (22.6 %)	
4	5 (15.2 %)	31 (12.3 %)	
5	3 (9.1 %)	37 (14.7 %)	
6	2 (6.1 %)	13 (5.2 %)	
7	9 (27.3 %)	43 (17.1 %)	0.35

For comparison between groups Fisher’s Exact test was used for ichotomous variables and the Mantel-Haenszel Chi Square Exact test was used for ordered categorical variables and the Mantel-Haenszel Chi Square test was used for ordered categorical variables and Chi Square Exact test was used for non-ordered categorical variables and Chi Square test was used for non-ordered categorical variables and the Mann-Whitney *U*-test was used for continuous variables

*PGP* pelvic girdle pain, *LBP* Low back pain, *RCT* Randomized controlled trial, *BMI* Body mass index, *P4-test* Posterior pelvic pain provocation test, *ASLR-test* Active straight leg test, *VAS* visual analoge scale. *DRI* Disability Rating Index; *EQ-5D* European Quality of Life measure – five dimensions; *EQ-VAS* European Quality of Life measure – visual analog scale. For categorical variables n (%) is presented. For continuous variables Mean (SD) / Median (Min; Max) / *n* = is presented

Table 2 shows the characteristics of the study population at follow-up. Women with PGP worked significantly fewer hours/week compared to women with no PGP at follow-up. In addition, sleep was disturbed in 26/37 (70 %) women, and 19/35 (54 %) women reported that their PGP was affected by the menstrual cycle (data not shown).

**Table 2:** Characteristics of women with PGP or PGP plus LBP and women with no PGP at follow-up

Variable	PGP or PGP + LBP (*n* = 37)	No PGP (*n* = 290)	p-value
Age, years	37.7 (4.9)	39.4 (5.7)	0.059
	38.0 (29.0; 49.0)	40.0 (24.0; 54.0)	
	*n* = 37	*n* = 290	
Time from randomisation to follow-up (years)	7.35 (4.02)	8.41 (3.80)	0.27
	5.00 (2.00; 12.00)	11.00 (1.00; 13.00)	
Working hours/week	35.7 (7.6)	37.1 (6.5)	0.032
	38.0 (19.0; 60.0)	40.0 (1.0; 49.0)	
	*n* = 29	*n* = 221	
Marital status			
Single	3 (8.6 %)	24 (9.5 %)	
Married/cohabitating	32 (91.4 %)	229 (90.5 %)	1.00
Number of pregnancies			
1	4 (12.1 %)	18 (7.2 %)	
2	8 (24.2 %)	89 (35.6 %)	
3	15 (45.5 %)	69 (27.6 %)	
4	6 (18.2 %)	73 (29.2 %)	
5	0 (0.0 %)	1 (0.4 %)	0.56
Parity			
1	5 (14.7 %)	30 (12.0 %)	
2	20 (58.8 %)	139 (55.4 %)	
3	8 (23.5 %)	75 (29.9 %)	
4	1 (2.9 %)	6 (2.4 %)	
5	0 (0.0 %)	1 (0.4 %)	0.52
Caesarean section	4 (14.3 %)	48 (19.0 %)	0.76
Birthweight last born baby, grams	3633 (492)	3692 (490)	0.43
	3548 (2995; 4615)	3700 (2129; 5000)	
	*n* = 30	*n* = 274	
Sex of last born baby, boy	15 (50 %)	140 (51 %)	0.925
	*n* = 30	*n* = 275	

For comparison between groups Fisher’s Exact test was used for dichotomous variables and the Mantel-Haenszel Chi Square Exact test was usedfor ordered categorical variables and the Mantel-Haenszel Chi Square test was used for ordered categorical variables and Chi Square Exact test was used for non-ordered categorical variables and Chi Square test was used for non-ordered categorical variables and the Mann-Whitney

*U*-test was used for continuous variables

*PGP* pelvic girdle pain, *LBP* Low back pain, For categorical variables n (%) is presented. For continuous variables Mean (SD) / Median (Min; Max) / n = is presented

Table 3 shows results of measurements of function (DRI, ODI), HRQL (EQ-5D, EQ-VAS, anxiety and depression (HADS), and pain catastrophizing (PCS) in women with PGP and women with no PGP at follow-up. Women classified with PGP had a significantly decreased ability to perform daily activities, lower self-efficacy, and decreased HRQL compared to women without PGP. Moreover, they had significantly higher levels of anxiety, depression and pain catastrophizing than women with no PGP. The time interval between original randomisation and follow-up did not impact the outcome measures assessed.

**Table 3:** Function (ODI), health related quality of life (EQ-5D, EQ-VAS), Anxiety and depression (HADS) and pain catastrophisation (PCS) in women PGP or PGP plus LBP and women with no PGP at follow-up

Variable	PGP or PGP + LBP (*n* = 37)	No pain (*n* = 290)	p-value
ODI	22.0 (6; 42)	4.00 (0; 58)	<.001
	*n* = 31	*n* = 243	
EQ-5D	0.725 (0.414; 1)	0.848 (0.222; 1)	<.001
	*n* = 25	*n* = 234	
EQ -VAS	76.5 (50; 100)	86.0 (0; 100)	<.001
	*n* = 30	*n* = 230	
HADS-A, sum of scores	66 (0; 14)	3 (0; 22)	<.001
	*n* = 32	*n* = 241	
HADS-A > 8	10 (31.3 %)	24 (10.0 %)	0.0044
HADS-D, sum of scores	2.50 (0; 12)	1.00 (0; 12)	<.001
	*n* = 32	*n* = 241	
HADS-D, >8	2 (6.3 %)	4 (1.7 %)	0.30
PCS score	15.5 (0; 35)	6 (0; 52)	<.001
	*n* = 32	*n* = 237	
PCS magnification	3.50 (0; 8)	1 (0; 12)	<.001
	*n* = 32	*n* = 241	
PCS helplessness	6 (0; 17)	2 (0; 24)	<.001
	*n* = 32	*n* = 241	
PCS rumination	5.50 (0; 11)	2 (0; 16)	0.0036
	*n* = 32	*n* = 241	
GSE (half scale)	33.0 (24.0; 40.0)	35.0 (10.0; 40.0)	0.046
	*n* = 32	*n* = 239	
DRI index	30.8 (2.8; 78.6)	4.62 (0; 55.85)	<.001
	*n* = 32	*n* = 243	
DRI, Dressing	4 (0; 100)	1 (0; 26)	<.001
	*n* = 33	*n* = 241	
DRI, Outdoor walks	11 (0; 90)	1.50 (0; 71)	<.001
	*n* = 33	*n* = 242	
DRI, Climbing stairs	12 (0; 100)	2 (0; 77)	<.001
	*n* = 33	*n* = 243	
DRI, Sitting for a longer time	30 (0; 80)	3 (0; 90)	<.001
	*n* = 33	*n* = 243	
DRI, Get up from chair	13 (0; 100)	2 (0; 77)	<.001
	*n* = 33	*n* = 243	
DRI, Standing bent	39 (0; 100)	3 (0; 94)	<.001
	*n* = 33	*n* = 243	
DRI, Carrying a bag	22 (0; 97)	2 (0; 69)	<.001
	*n* = 33	*n* = 242	
DRI, Running	38 (1; 100)	3 (0; 100)	<.001
	*n* = 33	*n* = 243	
DRI, Light work	17 (0; 90)	2 (0; 79)	<.001
	*n* = 33	*n* = 243	
DRI, Turn in bed	49 (2; 100)	3 (0; 98)	<.001
	*n* = 33	*n* = 242	
DRI, Heavy work	47 (0; 98.)	4 (0; 86)	<.001
	*n* = 33	*n* = 242	
DRI, Lifting heavy	26 (2; 100)	3 (0; 75)	<.001
	*n* = 33	*n* = 243	
DRI, Sports	17 (0; 100)	2 (0; 65)	<.001
	*n* = 33	*n* = 243	

For comparison between groups Fisher’s Exact test was used for dichotomous variables and the Mann-Whitney *U*-test was used for continuous variables

*PGP* Pelvic girdle pain, *LBP* Low back pain, *ODI* Oswestry disability index, *EQ-5D* EuroQol-5 dimensions, *EQ-VAS* EuroQol-visual analogue scale, *HADS-A* Hospitality anxiety depression score-anxiety, *HADS-D* Hospitality anxiety depression score-depression, *PCS* Pain catastrophisation scale, *GES* General self-efficacy scale. For categorical variables n (%) is presented. For continuous variables Median (Min; Max) / n = is presented

**Fig. 2:**
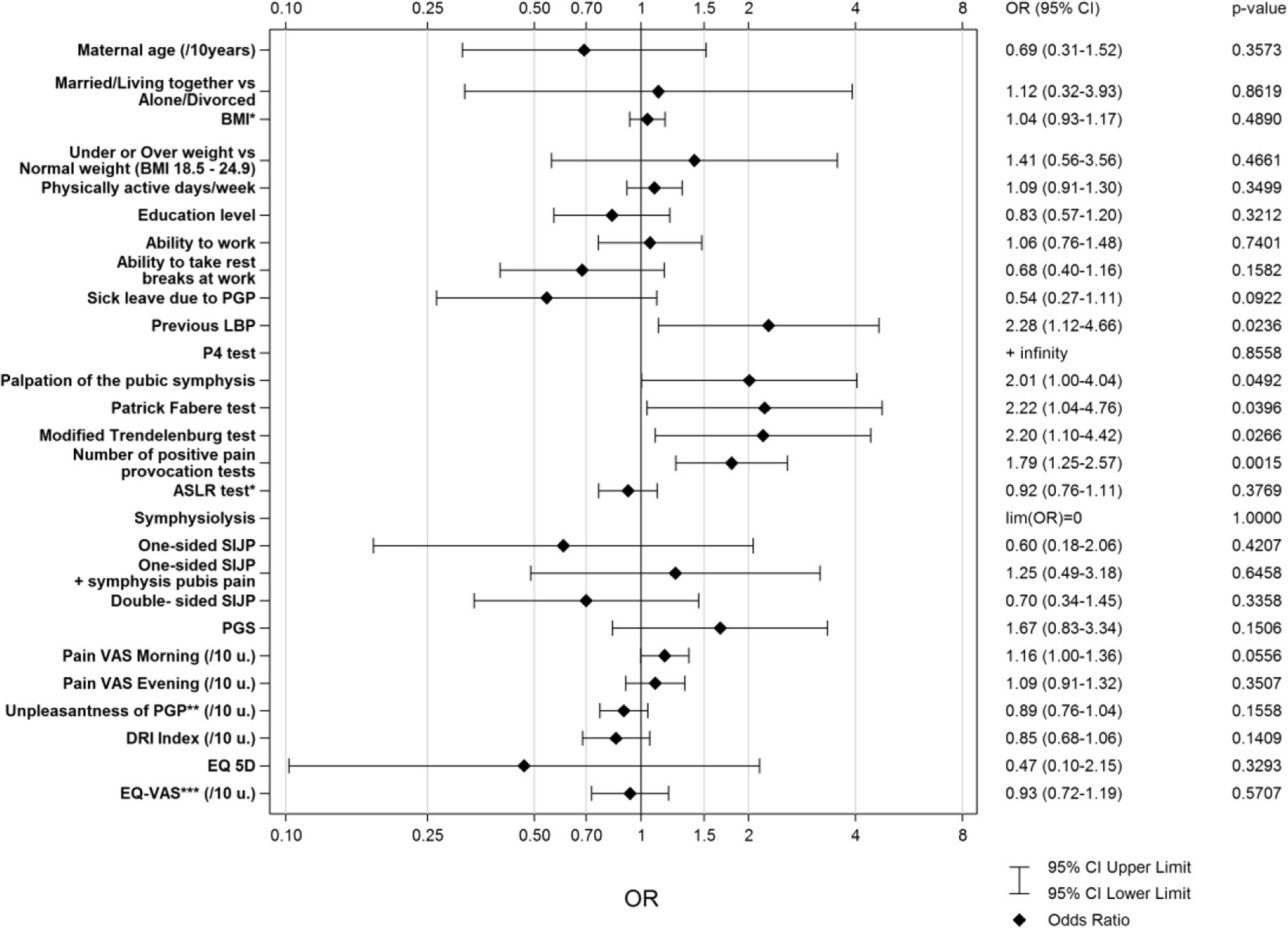
Association between baseline characteristics in pregnancy and the occurrence of PGP 2, 6 and 11 years after pregnancy. Result of the univariable logistic regressions with variables measured in pregnancy

Figure 2 shows the result of the univariable logistic regressions with variables measured in pregnancy from the 37 women with PGP compared to the results from the 290 women with no PGP. Earlier LBP (Odds Ratio, OR = 2.28), a positive symphysis pressure test (OR = 2.01), positive Faber (Patrick’s) test (OR = 2.22), a positive modified Trendelenburg test (OR = 2.20), and a high number of bilateral positive pain provocation tests (OR = 1.79) were predictors for long-term PGP. In the stepwise multivariable logistic regression the variable of a high number of bilateral positive pain provocation tests were entered into the model and resulted in an OR = 1.79 with a 95 % CI of 1.25-2.57 a P-value of 0.0015, and an area under the ROC-Curve of 0.65 with a 95 % CI of 0.55-0.75. After that no other variable was entered in to the model.

There was no association between characteristics registered at the follow-up such as: age at menarche, total number of pregnancies, parity, caesarean section (yes/no), birth weight (grams) or sex of last born baby and the occurrence of PGP 2, 6 and 11 years after pregnancy (data not shown).

## Discussion

### Main findings

Ten percent of women classified with PGP in pregnancy had PGP with considerable consequences on health and function in daily life up to a decade later. Pregnancy-related predictors for long-term PGP were: a history of LBP before index pregnancy, a high number of positive pain provocation tests, a positive symphysis pressure test, and a modified Trendelenburg or Patrick’s test. Some of the predictors were already known from shorter follow-up studies such as a history of LBP before pregnancy [2, 9, 27, 31] and a high number of positive pain provocation tests in pregnancy [31, 34], but Faber (Patrick's) test, the modified Trendelenburg’s and the symphysis pubic pressure tests as predictors are new findings. However, the Faber (Patrick's) test, as a predictor must be interpreted with caution since this is not a specific test for PGP*.*

In contrast to other follow-up studies [9, 12, 31], HRQL, decreased function, and high pain intensity were not shown to be significant pregnancy-related predictors. This may be explained by shorter follow-up periods in those studies, and that women with long-term PGP may have adjusted and confined their lives to decrease pain exacerbation. Also, a recently published syndrome-specific instrument may have been better for identifying PGP-specific functional limitations than the DRI [60]. That work-related factors such as strenuous work [28] and sick leave [29] were not confirmed as predictors may be interpreted by changes in work-related circumstances during the long time elapsed, and that sick leave is influenced by many factors, not only the severity of PGP. [19] Moreover, our study did not confirm age as a predictor for long-term PGP [23, 27]. However, age has been suggested as a bimodal factor due to conflicting results [2]. Neither did our study confirm that higher fetal weight [20] could predict long-term PGP. Nonetheless, the mean weight of the fetuses in the aforementioned study (3531 gram) was lower than the mean weight of the fetuses in both the PGP group and the non-PGP group in our study (3633, and 3692 grams, respectively). Moreover, other studies have [21, 26, 30] described an association between elective caesarean section and LBP 3 and 6 months after delivery, respectively. However, their results must be interpreted with caution because of a relatively small study sample [21], and that the reasons for caesarean delivery or vaginal delivery were not explored. Also, none of these studies used a medical examination for classification of PGP [20, 21, 26].

It is not surprising that long-term PGP, in common with many other chronic pain conditions, was associated with decreased HRQL, function, and psychosocial factors i.e. disturbed sleep, anxiety, depression, self-efficacy and pain catastrophizing [61, 62]. Anxiety and depression may act as facilitators of pain nociception, making the pain worse, and pain, in turn, may act as facilitators of anxiety and depression [63]. Sleep disturbance is also an important factor of chronic pain to identify and address [20]. However, as these factors were not measured in pregnancy no conclusions can be drawn regarding their predictive value and role in the development from acute to chronic PGP.

The results presented are also in line with earlier publications of persistent LBP and PGP in a shorter perspective. However, the prevalence of PGP was higher (10 %) in our study than in a 6-year postpartum follow-up study (7 %) [17]. The higher rate in our study can be explained by differences in the study populations, i.e. the women in our study may have had more severe PGP in pregnancy since they sought treatment, and that women participating in RCT 2-3 [45, 46] fulfilled the inclusion criteria of evening pain of at least 50/100. Two of the women had only symphysiolysis. Although this is a small number of women, there is a need to reconsider if women with isolated symphysiolysis recover completely as previously suggested [13].

### Strengths and limitations

There is a relative paucity of literature in this area. The topic is very important clinically, as pregnancy-associated musculoskeletal pain is highly prevalent and associated with significant morbidity that has a major socioeconomic impact on physical medicine as well as obstetric services.

The main strength of this work lies in that it is based on the follow-up of relatively robust randomised controlled trials, plus a relatively good response rate (71 %) from the original participants despite the time interval of up to 11 years. It is noteworthy that 26 of 37 respondents in the PGP group (70 %) and 213 of 290 respondents in the no PGP group (73 %) were in the active arms of the original trials, suggesting that fewer women randomised to the control arms (standard treatment only) completed the questionnaire. However, a majority of the women reporting LPP responded after the first questionnaire. None reporting LPP responded after reminder 2, which confirms the assumption that these women did not respond to the questionnaire because of the resolution of their LPP rather than neglect or other reasons. Prompt responses may also reflect the fact that many of these women receive insufficient care from healthcare provider’s barriers they encounter in seeking help [4, 64]. This can also be due to postpartum PGP being a neglected area of research. This is unacceptable both for the individual experiencing PGP and for the societal consequences of long-term pregnancy-related PGP. There was no evidence of bias caused by participant dropout on the prognostic factors or outcome; therefore, our findings are unlikely to be substantially influenced by loss at follow-up.

There is a potential for greater confounding in the group followed up at 11 years (the majority). These women are ultimately likely to be older and more likely to have had intercurrent pregnancies. However, there was no statistical significant difference in the time interval from randomisation to follow-up between women with PGP and women without PGP.

The reason for using both the ODI and the DRI for function was to be able to compare function measured in pregnancy with function measured at follow-up. The DRI was used in all the RCTs [44–46] but the ODI was added in Elden et al. 2008 [45] and Elden et al. 2013 [46] because some activities in the DRI e.g. running, lifting heavy objects and participating in sports was found inappropriate for women with PGP. The topics e.g. ability to care for oneself, ability to walk, ability to sit, sexual function, ability to stand, social life, sleep quality, and ability to travel in the ODI complements the DRI. The topics reflect both decreased body functions, activities in daily life and symptoms (pain), which are included in the European Guidelines of diagnosis and treatment for PGP [5] and reported in both quantitative [65] and qualitative studies describing women’s reported difficulties of life by women with PGP [8, 66–68] and their partners [69].

Other major strengths of this study are the relatively large sample of women classified with PGP in pregnancy [5] and baseline registration of pain and function before inclusion in the RCTs. In addition the examination of women with self-reported LPP was performed by skilled physiotherapists using the same classification at follow-up as in pregnancy, and the wide range of predictors measured by validated instruments. Another strength was that false positive pain provocation tests at follow-up were minimized by the identification of lumbar pain and the centralization phenomenon [70]. Furthermore, the use of pain provocation tests minimized false negative cases [7].

Other strengths are that many of the instruments used in this study have shown good internal consistency, test-retest reliability, and construct validity when used with a sample of participants with PGP postpartum [12, 17, 37, 71].

A weakness of our study was that the sample size (in the women with PGP, *n* = 37) is not of sufficient number to rule out a type II error for the small difference in the primary and secondary outcome variables measured. Moreover, BMI, breastfeeding and emotional distress shown to influence the prognosis of PGP, were not included [16, 24, 25, 35]. It may also be the case that predictors of PGP outcome are not fixed at specific time points (i.e. baseline registration before inclusion in an RCT) but are more fluid in nature and change, and evolve during different periods of the women’s experiences of PGP. Future studies with more frequent follow-ups will better identify such patterns. In addition, the study design could not control for other potential comorbid health conditions that may be accounting for an individual's PGP. Although care was taken to exclude any patients with current systemic disease (*n* = 11), the possibility of other conditions being present at some stage in the intervening years remains. Although parity did not differ significantly between participants with PGP versus those without PGP at follow-up, it remains unclear whether or not any of these intervening pregnancies were complicated by musculoskeletal pain conditions and whether there may have been a differential effect between the women with or without PGP. We have earlier shown that 99 % of women in the RCT performed 11 years ago [72] reported resolution of their pain by 12 weeks. Therefore pain reported 11 years later in these women may be new-onset (i.e. have nothing to do with the index pregnancy), or may indicate recurrence, i.e. is unlikely to reflect persistent pain. However, the low prevalence shown 12 weeks after pregnancy [72] might have reflected that the women had not yet resumed with their daily activities. To definitively associate the presence of PGP 11 years later with the index pregnancy without careful review of relevant events in between would potentially be misleading. Thus, the generalizability (external validity) of the study results may be limited to this population of women.

### Interpretation

PGP is closely related to pregnancy, and most commonly debuts in pregnancy. Thus, the debut of PGP is easy to identify and screen. Based on the results of our study we suggest that the classification of pregnant women with self-reported LPP include an examination of both the lumbar spine and the pelvic girdle, and that the P4 test is used to identify women with posterior pelvic pain, and the MAT-test for anterior pelvic pain, as these tests are reliable and valid for posterior and anterior PGP [5, 7, 55]. In additon, more pelvic pain provocation tests can be used to identify women with severe PGP, which in turn will indicate that these women run a higher risk of long-term PGP. The women should thereafter be offered structured and individualised treatment depending on the classification of their condition. Promising interventions for PGP during pregnancy are acupuncture, a semi-elastic belt, and specific stabilizing exercises [1, 44, 73]. Since it has been shown that improvement from persistent PGP level off around 6 months postpartum [74], It is of great importance that these women are followed up. Women that are not symptom free by that time most probably need individualised treatment depending on the classification of their condition in order to prevent long term pain [75, 76].

## Conclusions

In conclusion, this unique long-term longitudinal study highlights the importance of assessment and classification of LPP in pregnancy and at postpartum follow-up for the prediction of long-term PGP. One of ten women classified with PGP during pregnancy have PGP with severe consequences not only on women’s everyday life but also on their family life and economy up to 11 years later. Previous LBP and a high number of positive pain provocation tests are pregnancy-related predictors lasting more than a decade. The severity of PGP in pregnancy thus seems important to identify early, which is possible in most countries since these women are in frequent contact with the health care system. More attention should be paid to pregnant women with a history of LBP and many positive pain provocation tests and women with remaining PGP at follow-up postpartum. Focus should be to minimize the symptoms and consequences of PGP for the women. Further research of this major health problem is needed.

## Abbreviations

ASLR, Active Straight Leg Raising; BMI, Body Mass Index; LBP, Low Back Pain; LPP, Lumbopelvic pain; OR, Odds Ratio; P4 test, The posterior pelvic pain provocation test; PGP, Pelvic Girdle Pain; PGS, Pelvic Girdle Syndrome; RCT, Randomised Controlled Trial; SIJ, Sacroiliac joints.
